# Supplementary material for: Lansoprazole Increases Inorganic Pyrophosphate in Patients with Pseudoxanthoma Elasticum: A Double-Blind, Randomized, Placebo-Controlled Crossover Trial
Source: Int J Mol Sci. 2023 Mar 3;24(5):4899. doi: 10.3390/ijms24054899 (PMC10003519; doi:10.3390/ijms24054899)
Supplement: Supplementary file 1 [file ijms-24-04899-s001.zip › ijms-2175654-supplementary.pdf]

SUPPLEMENTARY MATERIAL

Table S1. Physical examination

Body weight (kg)	72.7 ±14.94
Height (cm)	1.62 ± 0.06
Body-mass index (kg/m^2)	26.19 ± 8.43
Waist circumference (cm)	96 ± 11.47
Systolic blood pressure (mmHg)	127.25 ± 18.50
Diastolic blood pressure (mmHg)	78.70 ± 9.21

Data are shown as n (%) or mean ± SD.

Table S2. Laboratory parameters in the final visits of each phase

Values in both final visits			
	**Placebo**	**Lansoprazole**	***p*-value**
Hemoglobin (g/dL)	14.16 ± 1.32	14.16 ± 1.17	0.9896
Hematocrit (%)	42.83 ± 3.68	43.33 ± 3.69	0.2186
Platelets (per mm^3)	236 .2 ± 48.59	245.1± 47.30	0.1527
Neutrophils (per mm^3)	4.07 ± 0.97	4.53 ± 1.11	0.4195
Prothrombin time (sc)	102.1 ± 15.6	100.7 ± 12.3	0.3273
aPTT (sec)	25.52 ± 1.76	27.97 ± 17	0.5041
Thrombin (sc)	11.53 ± 0.8	11.49 ± 0.51	0.9371
Ferritin (ng/mL)	58.11 ± 45.41	96.05 ± 97.10	0.2807
C-reactive protein (mg/L)	4.43 ± 1.88	4.36 ± 1.60	0.3305
Glucose (mg/dL)	103.68 ± 21.07	104.21 ± 24.31	0.9140
Urea (mg/dL)	34.74 ± 9.92	34.42 ± 10.39	0.8410
Creatinine (mg/dL)	0.81 ± 0.13	0.82 ± 0.14	0.8025
Calcium (mg/dL)	9.62 ± 0.39	9.81 ± 0.34	0.1341
Phosphorus (mg/dL)	3.59 ± 0.54	3.71 ± 0.49	0.4579
Uric acid (mg/dL)	4.37 ± 2.07	5.07 ± 1.62	0.2242
Cholesterol (mg/dL)	172 ± 40.49	182 ± 48.53	0.0747
HDL cholesterol (mg/dL)	56.73 ± 16.47	58.26 ± 19.08	0.4611
LDL cholesterol (mg/dL)	95.74 ± 35.2	107.36 ± 41.8	0.1126
Triglycerides (mg/dL)	115.8 ± 75.3	117.16 ± 60.7	0.9234

Abbreviations: aPTT, activated partial thromboplastin time; HDL, high-density lipoprotein; LDL, low-density lipoprotein. Data are showed as mean ± SD.

Table S3: PPi levels at four visits in the clinical trial

VISITS			
**PPi1**	**PPi2**	**PPi3**	**PPi4**
0,287	0,371	0,384	0,588
0,235	0,357	0,244	0,403
0,432	0,401	0,370	0,530
0,554	0,624	0,680	0,577
0,541	0,655	0,531	0,451
0,407	0,443	0,424	0,553
0,393	0,519	0,485	0,504
0,245	0,213	0,290	0,275
0,523	0,404	0,339	0,611
0,402	0,271	0,270	0,379
0,298	0,232	0,246	0,193
0,410	0,416	0,345	0,321
0,296	0,264	0,275	0,257
0,526	0,436	0,642	0,421
0,480	0,335	0,463	0,636
0,340	0,231	0,217	0,285
0,283	0,256	0,227	0,252
0,192	0,240	0,251	0,253
0,539	0,315	0,332	0,384
0,364	0,353	0,334	0,426

Individual data of PPi, µM

Table S4. Adverse events during clinical trial by study phase

**Placebo**	**Lansoprazole**
Dyspepsia (2 cases)<br>Gastric intolerance (drop out)	Dyspepsia (2)<br>Diarrhea (1)
Inguinal hernia<br>Back pain after a car accident<br>Sciatalgia	
